# Association of Taiyin personality traits with sleep quality among college students: parallel indirect effects through resilience and rumination

**DOI:** 10.3389/fpsyg.2026.1854838

**Published:** 2026-09-30

**Authors:** Guanghua Pan, Rui Zhang, Xiaokang Wang, Yuelin Zhai, Anran Wang, Haijuan Zhu

**Affiliations:** 1 College of Health Science, Shandong University of Traditional Chinese Medicine, Jinan, China; 2 Student Affairs Department, Shandong University of Finance and Economics, Jinan, Shandong, China; 3 School of Teacher Education, Qilu Normal University, Jinan, China

**Keywords:** parallel mediation, psychological resilience, ruminative thinking, sleep quality, Taiyin personality

## Abstract

**Aim:**

To investigate the relationships and pathways among Taiyin personality trait, resilience, rumination, and sleep status in a college student population.

**Methods:**

Using a cluster sampling method, 500 undergraduate students from a general higher education institution were selected as participants. They were surveyed using the Five-Pattern Personality Inventory (Taiyin Personality Subscale), the Rumination Response Scale, the Chinese Revised Version of the Connor-Davidson Resilience Scale, and the Pittsburgh Sleep Quality Index (Chinese Revised Version).

**Results:**

(1) The anal revealed a significant positive correlation between Taiyin personality and the Pittsburgh Sleep Quality Index score (*r* = 0.405, *p* < 0.01), and this personality trait positively predicted sleep quality (*β* = 0.130, *p* < 0.01). Individual scores on the Taiyin personality trait showed a positive correlation with Pittsburgh Sleep Quality Index scores, indicating that higher scores on this personality trait were associated with poorer sleep quality. (2) After controlling for all demographic variables, the Bootstrap test results confirmed that resilience played a partial mediating role between the Taiyin personality trait and the Pittsburgh Sleep Quality Index score. The mediation effect size was 0.0345 (95% CI: 0.015 to 0.054), accounting for 26.46% of the total effect. Rumination also served as a partial mediator between the Taiyin personality trait and the Pittsburgh Sleep Quality Index score, with a mediation effect size of 0.0567 (95% CI: 0.036 to 0.081), contributing 43.48% of the total effect.

**Conclusion:**

The Taiyin personality trait is directly correlated with the sleep status of college students and also indirectly influence sleep quality through the two pathways of resilience and rumination.

## 1 Introduction

Sleep is an indispensable life activity for the human body, and its quality serves as a crucial indicator for measuring an individual’s physical and mental health. College students represent a key reserve force for social construction and development, and their physical and mental development deserves focused attention from all sectors of society. According to data from the 2020 China College Student Health Survey Report, approximately 77% of surveyed college students reported experiencing sleep problems to varying degrees. Furthermore, findings from the 2025 China Sleep Health Research White Paper indicate that only 21% of college students manage to fall asleep before midnight, while 25% delay their bedtime until after 2 a.m. Additionally, 32% of students do not get out of bed until after 9 a.m. Insufficient sleep may lead to weight gain, mood deterioration, and even increase suicide risk by exacerbating depressive symptoms. Therefore, improving the sleep quality of college students is crucial for both individuals and society as a whole.

Traditional Chinese medicine theory posits that the vital functioning of the human body is grounded in the principles of yin-yang, qi, and blood. It advances the principle that “when yin is stabilized, and yang is secured, the spirit is well-regulated; when yin and yang are separated and severed, the spirit dissipates, “attributing the emergence of various bodily disorders to yin-yang imbalance. The classic medical text Ling Shu Jing categorizes personalities into five types based on an individual’s constitutional characteristics and behavioral manifestations: the sun-type, the lesser yang, the balanced yin-yang, the lesser yin, and the Taiyin personality.

Later, physicians have conducted five-pattern syndrome differentiation and treatment according to individuals’ physical features and behaviors. Except for those with balanced yin-yang, the other four patterns all involve a degree of yin-yang imbalance, with the degree being particularly pronounced in individuals exhibiting the sun-type personality and the Taiyin personality (Lu and Ya, 2016). The Ling Shu·Tong Tia describes the Taiyin type as “greedy and unkind, superficially amiable yet deep, fond of taking in and averse to giving out, with inner harmony that is not expressed, not attending to the times, and acting only after others.” Scholars have summarized these characteristics as a predominantly introverted personality, relatively unstable emotions, a significant tendency toward self-restraint and repression, and a slow but sensitive reaction to external stimuli. Furthermore, individuals with the Taiyin personality tend to be overly focused on their internal state, exhibit weaker adaptability in new environments, and display reserved and restrained behavior.

Simultaneously, this personality type is characterized by deep and meticulous thinking, a propensity for association, prominent imaginative abilities, and a proficiency in reflection and self-awareness. They are often accompanied by inner sensitivity and excessive worry, passive withdrawal and timidity when facing decisions, and cautious restraint. In social situations, they typically show low willingness to engage with others, a susceptibility to feelings of loneliness, and insufficient self- confidence (Yang and Xue, 2006). This personality trait manifests not only in individual ability development and daily life but also indirect effect physiological health. Individuals with the Taiyin personality, characterized by “predominant yin without yang, “are prone to negative emotions such as loneliness and withdrawal, which can subsequently correlate to issues like insomnia and insufficient sleep. Research indicates that compared to groups with other personality traits, these individuals are more sensitive to external stressors and exhibit particularly prominent sleep problems (Tang, 2015).

The stronger the level of the Taiyin Personality Trait, the more pronounced an individual’s insomnia symptoms typically become. Furthermore, traditional Chinese medicine constitutionology points out that patients with a yang-deficiency constitution commonly experience sleep quality issues, and yang deficiency is also the core constitutional feature of the Taiyin personality (Ha et al., 2024; Li et al., 2021). Based on this, this study proposes. *Hypothesis 1*: Higher Taiyin personality scores are associated with higher PSQI scores, indicating poorer sleep quality.

Specifically, a higher score on this personality dimension is associated with an increase in the sleep quality index score, indicating poorer sleep quality.

Psychological resilience refers to an individual’s ability to effectively adapt and return to normal functioning when confronted with adversity, trauma, or high-pressure situations (Masten, 2009). As a capacity for coping with stress and negative circumstances, psychological resilience is undoubtedly significant for the generation and maintenance of positive emotions. The broaden-and-build theory of positive emotions posits that positive emotions can promote psychological and physical health through more effective coping strategies (Fredrickson, 1998; Fredrickson, 2001). Consequently, research has demonstrated that psychological resilience is a core element in an individual’s stress coping and the maintenance of mental health. Park and Folkman (1997) noted that individuals who can find meaning in stressful events tend to possess stronger coping and adaptive abilities, which in turn lead to more positive psychological outcomes, such as improved sleep quality. As a protective factor for positive emotions, psychological resilience can effectively influence an individual’s sleep quality, thereby promoting physical and mental health (Tugade and Fredrickson, 2004; Lazarus and Folkman, 1984). Li et al. (2025) and Jiang et al. (2023) further found that psychological resilience serves as a protective factor for improving sleep quality. Additionally, multiple studies indicate a close correlation between personality traits and psychological resilience (Li et al., 2021; Fang et al., 2023; Sun et al., 2025). Building on this, Liu et al. (2022) discovered a significant correlation between the five-state personality types of nursing interns and their psychological resilience, with the Taiyin personality showing a negative association with psychological resilience. Based on the above findings, this study proposes. *Hypothesis 2*: The association between Taiyin personality and PSQI score shows a significant statistical indirect effect through psychological resilience.

Rumination denotes a cognitive response pattern in which an individual, when experiencing a Rumination is a negative emotional state that persistently focuses on the emotion itself, its origins, personal reactions, and possible adverse outcomes, without taking constructive or effective steps to address the issue (Han and Yang, 2009). Some researchers define this cognitive pattern differently. For example, Conway et al. (2000) describes it as a personality characteristic marked by continual negative thinking, the repeated generation of adverse thoughts, and difficulty disengaging from distressing emotions and challenges. This pattern reduces the drive for social engagement and obstructs adaptive behaviors. In traditional Chinese medicine theory, rumination shares characteristics with the Taiyin personality, such as gloom, reserve, and inhibition, which are described as negative personality traits (Zhang, 2009); the Taiyin personality is considered a subtype of negative personality. Based on this, it can be inferred that individuals with a Taiyin personality may be more prone to rumination and to focusing more on negative emotions. Empirical evidence has consistently shown a significant negative correlation between rumination and an individual’s sleep quality (Baglioni et al., 2010; Pilcher and Huffcutt, 1996). Studies on various populations, such as elderly parturients and college students, have confirmed this relationship (Liang, 2025; Zheng et al., 2025). According to the cognitive model of insomnia, when an individual engages in rumination before sleep, it induces a higher level of cognitive arousal, which subsequently adversely affects sleep quality (Tousignant et al., 2019). The perseverative cognition hypothesis proposed by Brosschot et al. (2006) further explains that rumination maintains a state of sustained psychological and physiological activation, causing both short-term and long-term disruptions to sleep. Therefore, this study proposes *Hypothesis 3*: The association between Taiyin personality and PSQI score shows a significant statistical indirect effect through rumination. While existing studies have addressed the pairwise relationships between Taiyin personality, psychological resilience, rumination, and sleep quality, few have integrated these factors for a comprehensive examination. This study aims to examine how Taiyin personality is related to sleep quality. It also explores the mediating roles of psychological resilience and rumination. These insights will provide theoretical support and practical references for intervention pathways to improve sleep quality among college students.

Although previous research has examined the mediating roles of psychological resilience and rumination between personality traits and sleep quality, whether these two variables operate as independent parallel paths or as serial mediators remains theoretically underexplored. In the present study, we adopt a parallel mediation framework based on both theoretical and empirical considerations. Theoretically, the core features of Taiyin personality—deep introspection, interpersonal sensitivity, and internalized distress—may exert dual and relatively independent influences: on one hand, they undermine individuals’ capacity for flexible adaptation and positive coping, thereby hindering the development and maintenance of psychological resilience; on the other hand, they heighten cognitive reactivity to negative emotional experiences and foster a perseverative focus on internal states, thereby amplifying ruminative thinking. This suggests that Taiyin personality is not necessarily related to one regulatory process first and then subsequently influence the other; rather, it can simultaneously have an effect on both adaptive and maladaptive regulatory pathways. From the perspective of Lazarus and Folkman's (1984) cognitive appraisal theory, when individuals encounter interpersonal and internal stressors derived from Taiyin traits, problem-focused coping (closely tied to resilience) and emotion-focused coping (such as rumination) can be co-activated, without an inevitable causal ordering between them. Empirically, the weak negative correlation observed between psychological resilience and rumination in our preliminary analysis (*r* = −0.289) further supports their relative independence in this population, providing initial statistical justification for a parallel specification. Importantly, however, we explicitly acknowledge that the present study was designed as a parallel mediation model and did not test the alternative serial mediation path (i.e., Taiyin personality → psychological resilience → rumination → sleep quality). The parallel framework adopted here is a theoretically driven choice, and future research should conduct direct empirical comparisons to examine under what conditions these two mechanisms operate independently or sequentially.

## 2 Research subjects and methods

### 2.1 Research subjects

This study employed a cluster sampling method to conduct a questionnaire survey among university students in Jinan and Tai’an between June and September 2025. Data collection utilized a combination of online and paper questionnaires. The participants were undergraduate students aged 16 to 24. All participants took part voluntarily, completed the questionnaires anonymously, and signed informed consent forms.

In this study, a total of 500 questionnaires were distributed, with 459 returned. After excluding invalid responses, 401 valid samples were obtained, yielding an effective response rate of 87.36%. The demographic characteristics of the sample are as follows: gender: 128 males (31.9%) and 273 females (68.1%); major: 142 students in arts (35.4%) and 259 in science and engineering (64.6%); household registration: 205 from rural areas (51.1%) and 196 from urban areas (48.9%); age: 52 individuals aged 16–18 (12.97%), 126 aged 19–20 (31.42%), 205 aged 21–23 (51.12%), and 18 aged 24 or above (4.49%). Regarding sample composition, there were 48 freshmen, accounting for 12.0%; 199 sophomores, 49.6%; 55 juniors, 13.7%; and 100 seniors, 24.9%. Regarding the distribution of only children, there were 257 non-only children (64.1%), 142 only children (35.4%), and 2 individuals (0.5%) with missing data for this item. Although the sample covered multiple grade levels, female participants (68.1%) and sophomores (49.6%) were overrepresented, which may limit the generalizability of the findings to male-dominant or other grade-level populations. To control for the potential confounding effects of gender, age, and grade level on the study results, these variables were included as control variables in the statistical model for the mediation effect analysis.

### 2.2 Research tools

#### 2.2.1 Five-Personality Test (Taiyin Personality Subscale)

This assessment tool was developed by Xue and Yang in 1988 and revised in 2008. It is China’s first independently revised localized personality assessment instrument. The scale consists of 103 items, comprising five subscales (Taiyang, Shaoyang, Yin-Yang Balance, Shaoyin, and Taiyin) and a lie scale. Higher scores on each dimension indicate a more pronounced personality tendency in that dimension. This study utilized the 2008 revised version of the Five-Personality Test with its national norm, which is based on a valid sample of 14, 486 cases. The instrument demonstrates good reliability and validity. For this study, the Taiyin Personality Subscale and the Lie Scale were used, with a Cronbach’s alpha coefficient of 0.985.

#### 2.2.2 Pittsburgh Sleep Quality Index

This study employed the Pittsburgh Sleep Quality Index (PSQI), originally developed by Buysse et al. (1989) and subsequently revised by Liu et al. (1996). This instrument encompasses seven components: subjective sleep quality, sleep onset latency, sleep duration, sleep efficiency, sleep disturbance, use of hypnotic medication, and daytime dysfunction. It consists of a total of 19 self- rated items and 5 items rated by others. The first 18 self-rated items are used to calculate scores for the seven components, each rated on a scale from 0 to 3. The total PSQI score is the sum of the scores from these seven components, with a lower total score indicating better sleep quality. The scale demonstrates good reliability and validity. In this study, Cronbach’s Alpha Coefficient was 0.796.

#### 2.2.3 Connor-Davidson Resilience Scale

This study employed the Connor-Davidson Resilience Scale (CD-RISC), originally developed by Connor and Davidson in 2003 and subsequently revised by Yu and Zhang (2007). The scale is designed to assess an individual’s psychological adaptability in the face of adversity, trauma, and stress. It consists of 25 items, which form three subscales: tenacity, self-reliance, and optimism. A 5-point Likert scale is used for scoring, where 1 indicates “strongly disagree” and 5 indicates “strongly agree.” Higher scores reflect a greater level of psychological resilience. The scale demonstrated a Cronbach’s alpha coefficient of 0.961 in this study.

#### 2.2.4 Ruminative Responses Scale

This study employed the Ruminative Responses Scale (RRS) revised by Han and Yang (2009). This instrument contains 22 items covering three dimensions: obsessive thinking, in-depth reflection, and repetitive symptom thinking. It uses a 4-point scoring method, where higher scores indicate a more severe tendency toward rumination in the individual. The Cronbach’s alpha coefficient for this scale in the present study was 0.964.

### 2.3 Data processing

Data analysis was conducted using SPSS 26.0 and the PROCESS macro. The test for mediation effect was performed with reference to the bias-corrected percentile Bootstrap method (Fang et al., 2012). A t-test was used to compare the differences between the Taiyin Personality of university students and the national norm. Pearson correlation analysis was employed to examine the relationships among Taiyin Personality, sleep quality, psychological resilience, and rumination.

## 3 Results analysis

### 3.1 Common method bias test results

Procedural measures, including anonymous questionnaire completion and lie-detection items, were used to reduce response-related bias. Harman’s single-factor test showed that the first unrotated factor accounted for 37.916% of the total variance, indicating that no single factor dominated the covariance structure. However, given the limitations of the Harman’s single-factor test as a purely statistical diagnostic (Podsakoff et al., 2003), we acknowledge that this method does not fully rule out common method bias. Nevertheless, the proportion is within an acceptable range, indicating that common method bias is unlikely to be a severe threat to the validity of our findings.

### 3.2 Descriptive statistics and correlation analysis

An independent samples t-test showed that the score on the Taiyin Personality dimension among university students (17.21 ± 6.40) was significantly higher than the national norm score for this dimension (9.38 ± 4.76) (*p* < 0.01). Nevertheless, caution is needed when interpreting this comparison, because our sample exhibited marked differences in gender composition relative to the norming sample. Correlation analysis indicated a significant positive correlation between Taiyin Personality and the Sleep Quality Index (*r* = 0.405, *p* < 0.01), as well as a significant positive correlation between Taiyin Personality and Rumination (*r* = 0.530, *p* < 0.01). Conversely, Taiyin Personality showed a significant negative correlation with Psychological Resilience (*r* = − 0.500, *p* < 0.01). Regarding the Sleep Quality Index, it was significantly negatively correlated with Psychological Resilience (*r* = −0.389, *p* < 0.01) and significantly positively correlated with Rumination (*r* = 0.462, *p* < 0.01). Furthermore, correlation analysis revealed a negative association between Psychological Resilience and Rumination (*r* = −0.289, *p* < 0.05), although the strength of this correlation was weak (|*r*| < 0.3). The results are presented in Table 1.

**Table 1:** Mean, standard deviation and correlation coefficient of each variable.

Variable	M	SD	1	2	3	4
1. Taiyin personality	17.21	6.40	1			
2. Psychological Resilience	86.63	15.299	−0.500**	1		
3. Rumination	43.92	13.467	0.530**	−0.289**	1	
4. Sleep Quality Index	9.12	3.088	0.405**	−0.389**	0.462**	1

*p < 0.05, **p < 0.01, ***p < 0.001.

### 3.3 Mediation effect analysis

This study constructed a dual-mediation effect model with Taiyin Personality as the independent variable, Sleep Quality as the dependent variable, and Psychological Resilience and Rumination as parallel mediating variables. To control for the potential confounding effects of gender, age, and grade on the core variables, these factors were included as covariates in the statistical model. The coding rules for these control variables were as follows: gender (male = 0, female = 1) and grade (freshman = 1, sophomore = 2, junior = 3, senior = 4). According to the research by Liu et al. (2022) these variables have a significant effect on sleep status of university students. Preliminary analysis results indicated that household registration and being an only child showed no significant association with Sleep Quality (*p* > 0.05); therefore, they were not included in the model. The mediation effect analysis indicated that Taiyin Personality had a significant positive predictive effect on the Pittsburgh Sleep Quality Index (*β* = 0.130, *p* < 0.01), supporting Hypothesis 1. Higher Taiyin personality scores are associated with higher PSQI scores, indicating poorer sleep quality. Specifically, a higher score on this personality dimension is associated with a higher score on the Pittsburgh Sleep Quality Index, indicating poorer sleep status. After introducing the mediating variables, Psychological Resilience and Rumination together explained an additional 12.51% of the variance in the Pittsburgh Sleep Quality Index attributable to Taiyin Personality (ΔR^2^ = 0.1251). The analysis results showed that Taiyin Personality had a significant negative predictive effect on Psychological Resilience (*β* = −0.763, *p* < 0.05), and Psychological Resilience significantly negatively predicted the Pittsburgh Sleep Quality Index (*β* = −0.0451, *p* < 0.05). Psychological resilience played a partial mediating role between the two, with its direct effect reaching a significant level (*β* = 0.039, *p* < 0.05). Taiyin Personality had a significant positive predictive effect on rumination (*β* = 0.712, *p* < 0.001), and rumination significantly and positively predicted the Pittsburgh Sleep Quality Index (*β* = 0.0796, *p* < 0.001). Rumination exhibited a partial mediating effect in the relationship between the two, with the direct path effect being significant (*β* = 0.039, *p* < 0.05). The specific results are detailed in Table 2.

**Table 2:** Analysis of the mediating effect of mental resilience and rumination.

Predictor	Sleep Quality Index		Mental Resilience		Rumination		Sleep Quality Index	
	*β*	*(t)*	*β*	*(t)*	*β*	*(t)*	*β*	*t*
Grade	−0.083	−0.370	0.186	0.170	−0.453	−0.490	−0.038	−0.190
Age	0.181	1.910	−0.278	−0.610	−0.046	−0.120	0.172	1.970*
Gender	0.786*	2.08	−1.441	−0.790	−1.449	−0.930	0.836	2.390*
Taiyin Personality	0.130**	8.140	−0.763	−9.910**	0.712	10.840**	0.039	2.040*
Mental Resilience	—		—		—		−0.0451	−4.080**
Rumination	—		—		—		0.0796	6.140**
R	0.458		0.506		0.548		0.578	
R^2^	0.210		0.256		0.301		0.335	
F	9.940***		12.878***		16.122***		14.983***	

*p < 0.05, **p < 0.01, ***p < 0.001.

The results of the mediation effect test indicated that the total effect, the direct path, and the indirect path of the model were all significant, with none of the Bootstrap 95% confidence intervals for the corresponding paths containing zero. The total effect value of Taiyin Personality on the Pittsburgh Sleep Quality Index was 0.1304 (95% CI: 0.0989 to 0.1619). After introducing the mediating variables Psychological Resilience and Rumination, the direct effect value was 0.0392.

(95% C: 0.0014 to 0.0771). The combined mediation effect of Psychological Resilience and Rumination was 0.0912 (95% CI: 0.0628 to 0.1221), accounting for 69.94% of the total effect. A comparison of the two mediation effects revealed that the mediation effect of Psychological Resilience between Taiyin Personality and Sleep Quality Index accounted for 26.46% of the total effect, while the proportion explained by Rumination was 43.48%. The mediation strength of the latter was significantly higher than that of the former. Thus, research hypothesis 2 (that psychological resilience plays a mediating role in the relationship) and hypothesis 3 (that rumination plays a mediating role in the relationship) were both validated. Rumination mediates the relationship between Taiyin Personality and sleep quality among the college student population. The results are presented in Table 3, and the path analysis is shown in Figure 1.

**Table 3:** Decomposition table of total effect, direct effect, and mediating effect of Taiyin personality.

Path	Effect size	Boot standard error	95% CI	Relative effect size
Taiyin Personality → Sleep Quality Index	0.1304	0.016	0.0989 ~ 0.1619	100%
Taiyin Personality → Sleep Quality Index	0.0392	0.0192	0.0014 ~ 0.0771	30.06%
Taiyin Personality → mediating variable → Sleep Quality Index	0.0912	0.0152	0.0628 ~ 0.1221	69.94%
Taiyin Personality → Psychological Resilience → Sleep Quality Index	0.0345	0.0098	0.0153 ~ 0.0536	26.46%
Taiyin Personality → Rumination → Sleep Quality Index	0.0567	0.0116	0.0357 ~ 0.0814	43.48%

**Figure 1:**
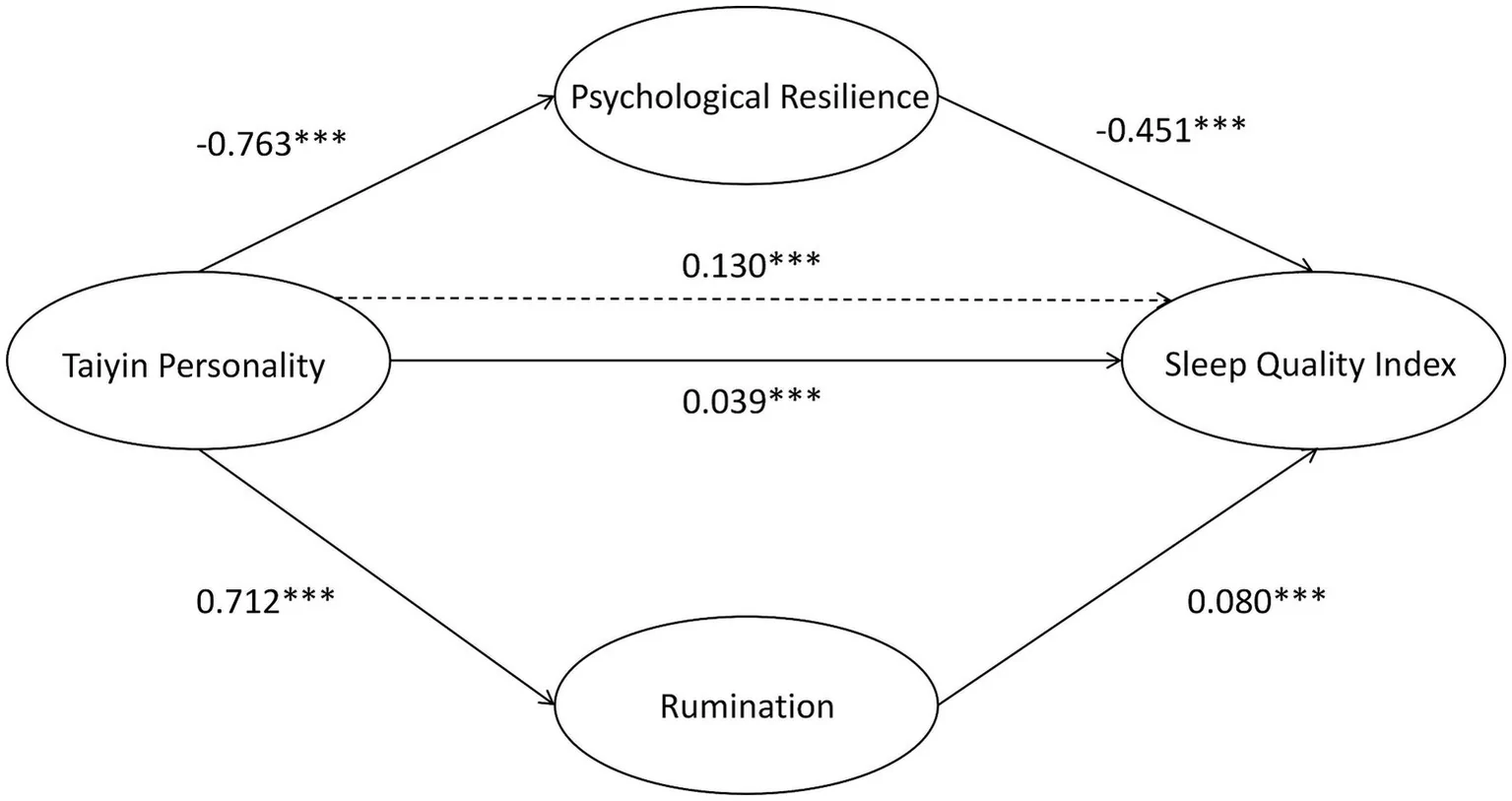
The dual mediation model of Taiyin personality’s influence on sleep quality (*N* = 401). The model has controlled for the effects of gender, age, and grade. *p < 0.05, **p < 0.01, ***p < 0.001.

## 4 Discussion

Based on cross-sectional survey data, this study explores the association between Taiyin personality trait and sleep quality among college students, and examines the statistical indirect effects of psychological resilience and rumination in this association. Given the cross-sectional design, the present findings do not permit causal inferences regarding the relationships among the variables. Within this boundary, the results indicate that Taiyin personality traits are significantly positively associated with poorer sleep quality, and that this association is partially transmitted through reduced resilience and heightened rumination. Specific relevant analysis results revealed a significant positive correlation between Taiyin personality and the Pittsburgh Sleep Quality Index, as well as between Taiyin personality and ruminative thinking. In contrast, a significant negative correlation was found between Taiyin personality and psychological resilience. These findings align with existing research (Liu et al., 2022; Tang, 2015). Simultaneously, a significant positive correlation was observed between psychological resilience and sleep quality, whereas ruminative thinking and sleep quality showed a negative correlation. In other words, individuals with higher levels of psychological resilience tend to have better sleep quality, while a stronger tendency toward ruminative thinking is associated with poorer sleep quality. It is noteworthy that this study found only a weak negative correlation between psychological resilience and ruminative thinking, which was insufficient to constitute a complete chain mediating effect in the model. This finding diverges considerably from numerous previous studies, which have consistently demonstrated a significant, strong negative correlation between the two variables. For instance, Chen and Chen (2025) found a significant, strong negative correlation (*r* = − 0.512) between psychological resilience and ruminative thinking among rural left-behind children. Similarly, Zhang et al. (2021) reported a significant negative correlation (*r* = −0.46) between psychological resilience and ruminative thinking among college students under stressful conditions. In light of this, the present study offers an in-depth discussion on this point. The underlying reason, we posit, is that the discrepancy between this result and those of previous studies may stem from the unique personality-level antecedent variable introduced in this research: the Taiyin personality. For instance, from the perspective of interpersonal interaction, the Taiyin personality trait is more likely to induce interpersonal stress, which is chronic and internalized. This may lead individuals to develop a specific psychological context. When such a negative psychological context serves as a stressor, it is difficult to resolve through direct action. Even individuals with high levels of psychological resilience may engage in ruminative thinking due to interpersonal distress—this persistent and ongoing interpersonal distress. Consequently, the correlation between the two diminishes, rendering them two independent, rather than interconnected, pathways for coping with the same stressor. Furthermore, according to the Cognitive Appraisal Theory of Stress proposed by Lazarus and Folkman (1984), when faced with difficulties stemming from Taiyin personality traits, if an individual appraises the situation as uncontrollable, they may still resort to the emotion-focused cognitive strategy of ruminative thinking to deal with the problem (Tugade and Fredrickson, 2004), even if they possess high levels of psychological resilience resources (Smith et al., 2008). However, it is important to emphasize that this interpretation is speculative and derives from our current correlational findings. Since our study was designed as a parallel mediation model and did not test a serial mediation path (Taiyin → resilience → rumination → sleep quality), we cannot draw definitive conclusions about the sequential relationship. This suggesting that the association pattern between the two variables may be context specific under Taiyin personality; future studies may construct serial mediation models for further comparison and verification.

This study found that Taiyin personality positively predicts the Pittsburgh Sleep Quality Index score, meaning that the more pronounced this personality trait is, the higher the score on the Pittsburgh Sleep Quality Index (which indicates poorer sleep quality). This result supports Hypothesis 1. The finding is consistent with the conclusion of Zhou's (2011) study on individuals with insomnia, which reported a significant negative association between Taiyin personality and sleep quality and further pointed out that both factors are highly correlated with insomnia. Tang (2015) further confirmed that the Taiyin personality factor significantly explains and predicts sleep disturbances. Individuals with Taiyin personality characteristics are more prone to sleep problems, and as the “yin” component within the personality traits increases, the incidence of sleep difficulties also rises. Based on the above analysis, Taiyin personality can be considered a significant risk factor for declining sleep quality. The deterioration in sleep quality may be associated with the characteristic manifestations of Taiyin personality, such as a pessimistic tendency, indecisiveness, excessive rumination, and cautious timidity, which are prominently observed in individuals with this personality type. Their highly introspective nature predisposes them to persistently reflect on negative events from daily life, particularly in academic and social contexts, during nighttime hours. This leads to elevated levels of brain arousal, subsequently triggering sleep difficulties, including trouble falling asleep, light sleep, and frequent dreaming.

This study confirms that psychological resilience plays a partial mediating role between Taiyin personality and sleep quality, supporting Hypothesis 2. The association between Taiyin personality and PSQI score shows a significant statistical indirect effect through psychological resilience.

This conclusion has been verified and supported by numerous previous studies. Scholars Li and Ding (2013) found that students with a tendency toward Taiyin personality are more likely to encounter difficulties in psychological adaptation. When facing adversity, they often exhibit pessimism, indecisiveness in decision-making situations, insufficient tolerance under pressure, and avoidance of difficulties when confronting risks, which weakens their ability to adapt to adversity and consequently lowers their level of psychological resilience. This study posits that psychological resilience, as a capability, is not only influenced by personality factors but can also have an effect on an individual’s physiological functioning. Serving as a protective factor (Guida et al., 2023), psychological resilience plays a moderating role in sleep quality. Numerous studies have reached consistent conclusions. There is a significant negative correlation between psychological resilience and the Pittsburgh Sleep Quality Index. Particularly among college students, psychological resilience demonstrates a significant negative predictive effect on the Pittsburgh Sleep Quality Index. This indicates that individuals with higher levels of psychological resilience tend to have better sleep quality (Dai et al., 2024; Li and Liu, 2023). This study posits that the synergistic development of an individual across multiple dimensions is jointly influenced by a multi-layered ecosystem. Bronfenbrenner and Ceci’s (1994) ecological systems theory suggests personality traits, as core elements of the individual system, can significantly impact physiological functions. These statistical predictions can indirectly affect sleep. As one such personality trait, psychological resilience can regulate the levels of stress hormones like cortisol, consequently reducing emotional volatility (Southwick and Charney, 2012). Individuals with high psychological resilience tend to employ problem-focused coping strategies and expand their psychological tolerance threshold, thereby mitigating the negative correlate of stress on sleep (Bonanno, 2004). The aforementioned research collectively demonstrates that college students with high psychological resilience are able to maintain adaptive behaviors, effectively buffer external pressures, reduce the incidence of anxiety and depression, and further improve their sleep quality.

This study found that rumination plays a partial mediating effect between Yin personality and sleep quality, meaning that Yin personality traits can reduce sleep quality by increasing the tendency toward rumination. The research also revealed that certain characteristics manifested in Yin personality traits bear a high degree of similarity to the manifestations of rumination. Moreover, this stable personality trait can, to some extent, influence the acquired cognitive style. Consequently, college students with a Yin personality often exhibit a pronounced tendency toward rumination. Furthermore, this study found that the tendency toward rumination is negatively related to individuals at a physiological level, not only generating negative emotions but also causing significant impairment to sleep quality. This finding aligns with the results of numerous other scholars. Brosschot’s Cognitive Hypothesis posits that rumination, as a sustained cognitive process, induces a state of psychophysiological activation, which in turn depletes cognitive resources and consequently interferes with normal sleep. The disruptive effect on sleep is particularly pronounced for symptom rumination (i.e., immersion in negative feelings) (Brosschot et al., 2006). Concurrently, the Associative Networks Theory indicates that rumination accelerates the activation of negative emotional coding, thereby intensifying emotional responses (Bower, 1981). Furthermore, rumination shows a significant positive correlation with levels of both anxiety and depression (Nolen-Hoeksema et al., 2008). These negative emotions can also lead to problems such as prolonged sleep latency, early morning awakening, and sleep architecture disturbance (Baglioni et al., 2011).

Overall, Taiyin Personality is not only directly associated with the sleep quality of college students but also exerts an indirect influence through two mediating pathways: psychological resilience and rumination. Specifically, the more pronounced an individual’s Taiyin Personality traits are, the lower their level of psychological resilience tends to be, and the poorer their sleep condition becomes. Simultaneously, a stronger tendency toward rumination is associated with a significant deterioration in sleep quality. Therefore, targeted interventions for college students with Taiyin Personality can be implemented from three aspects. First, enhance the psychological resilience of this student group. Schools can regularly organize Group Counseling sessions or Mental Health Salons focused on improving resilience to setbacks, helping students build this capacity and maintain an optimistic mindset in adversity, thereby effectively boosting their psychological resilience. On the other hand, it is essential to reduce the level of rumination in these college students. Universities can establish relevant Personal Growth Training Courses, supported by professional assistance, and incorporate programs such as Mindfulness-Based Cognitive Therapy and Mindfulness Relaxation. These initiatives can guide students to break free from negative thought cycles during the pre-sleep period, lower cognitive arousal levels, and thereby foster a favorable psychological environment for sleep. Furthermore, while Taiyin personality traits may serve as a useful research indicator for identifying individuals who could benefit from greater attention to sleep and emotion regulation, the current cross-sectional findings do not support using the Five-Pattern Personality Inventory as a routine screening tool in university settings. Such an application risks oversimplifying the complex causes of sleep problems and may inadvertently correlate to personality labeling. We recommend that practical efforts focus on non-stigmatizing interventions targeting sleep, resilience, and rumination, while positioning Taiyin personality solely as a research-oriented individual difference indicator. For students who exhibit high levels of Taiyin-related characteristics, voluntary support options—such as psychoeducational workshops or selective counseling referrals—may offer more ethically appropriate pathways. The effectiveness of such approaches warrants further validation through longitudinal or intervention studies in future research.

### 4.1 Limitations and future directions

At the same time, this study still has several limitations. First, due to its cross-sectional study design, it is difficult to establish clear causal relationships among the variables. Future research is advised to adopt a longitudinal follow-up design to verify the temporal characteristics of the mediation effect ad to explore causal relationships. Second, the sample consisted solely of college students from Jinan ad Tai’an, which did not adequately account for the influence of regional and cultural differences. Third, all variables were measured via self-report questionnaires at a single time point. Although Harman’s single-factor test suggested no substantial common method bias, this statistical approach has been criticized for its low sensitivity (Podsakoff et al., 2012). Future research could adopt multi-source data (e.g., peer- or teacher-reported resilience) or time-lagged designs to further reduce the potential inflation of relationships due to shared method variance. Additionally, the gender and grade distributions in our sample were unbalanced, with a higher proportion of female and sophomore students. Given that gender has been consistently linked to personality traits and sleep patterns in college samples, caution is warranted when generalizing the present results to male students or students at other academic stages.” Future studies with more balanced demographic recruitment are warranted. Furthermore, the age of the participants was concentrated between 16 and 24 years, lacking representation form a broader age range. Subsequent studies should aim to expand the participant pool to include all age groups and a nationwide scope to reduce the correlate of such differences. Additionally, the data primarily relied on student self-assessment. Future research should, where possible, collaborate with mental health educators to conduct rater-rated scale assessments to obtain more objective data. This is of significant importance for advancing research on the relationship between TCM Personality Theory and physical and mental data availability statement.

The datasets generated during this study are available from the corresponding author on reasonable request.

## Data Availability

The original contributions presented in the study are included in the article/Supplementary material, further inquiries can be directed to the corresponding author.

## References

[ref1] Baglioni C. Battagliese G. Feige B. Spiegelhalder K. Nissen C. Voderholzer U. . (2011). Insomnia as a predictor of depression: a meta-analytic evaluation of longitudinal epidemiological studies. J. Affect. Disord. 135, 10–19. doi: 10.1016/j.jad.2011.01.011, 21300408

[ref2] Baglioni C. Spiegelhalder K. Lombardo C. Riemann D. (2010). Sleep and emotions: a focus on insomnia. Sleep Med. Rev. 14, 227–238. doi: 10.1016/j.smr.2009.10.007 20137989

[ref3] Bonanno G. A. (2004). Loss, trauma, and human resilience: have we underestimated the human capacity to thrive after extremely aversive events? Am. Psychol. 59, 20–28. doi: 10.1037/0003-066X.59.1.20, 14736317

[ref4] Bower G. H. (1981). Mood and memory. Am. Psychol. 36, 129–148. doi: 10.1037/0003-066X.36.2.129, 7224324

[ref5] Bronfenbrenner U. Ceci S. J. (1994). Nature-nurture reconceptualized in developmental perspective: a bioecological model. Psychol. Rev. 101, 568–586. doi: 10.1037/0033-295X.101.4.568, 7984707

[ref6] Brosschot J. F. Geri W. Thaye J. F. (2006). The perseverative cognition hypothesis: a review of worry, prolonged stress-related physiological activation, and health. J. Psychosom. Res. 60, 113–124. doi: 10.1016/j.jpsychores.2005.06.074, 16439263

[ref7] Buysse D. J. Reynolds C. F. III Monk T. H. Berman S. R. Kupfer D. J. (1989). The Pittsburgh sleep quality index: a new instrument for psychiatric practice and research. Psychiatry Res. 28, 193–213. doi: 10.1016/0165-1781(89)90047-4, 2748771

[ref8] Chen M. F. Chen L. X. (2025). The relationship between psychological resilience and rumination in rural left- behind children. J. Educ. Biol. 13, 376–380.

[ref9] Conway M. Csank P. A. Holm S. L. Blake C. K. (2000). On assessing individual differences in rumination on sadness. J. Pers. Assess. 75, 404–425. doi: 10.1207/S15327752JPA7503_04, 11117154

[ref10] Dai J. M. Zhu Y. J. Hu X. Y. Liu Z. H. Li Y. (2024). A study on the correlation between psychological resilience and sleep quality among college student athletes. Contemp. Sports Technol. 14, 179–182. doi: 10.16655/j.cnki.2095-2813.2024.26.049

[ref11] Fang J. Zhang M. Q. Qiu H. Z. (2012). Testing methods and effect size measurement for mediation effects: a review and prospect. Psychol. Dev. Educ. 28, 105–111. doi: 10.16187/j.cnki.issn1001-4918.2012.01.015

[ref12] Fang X. H. Zheng L. W. Rao Y. L. Zou L. Y. Lin C. X. Wang Y. J. (2023). The mediating role of psychological resilience between personality traits and active procrastination among nursing undergraduates. Occup. Health 39, 965–969. doi: 10.13329/j.cnki.zyyjk.20230321.002

[ref13] Fredrickson B. L. (1998). What good are positive emotions? Rev. Gen. Psychol. 2, 300–319. doi: 10.1037/1089-2680.2.3.300, 21850154 PMC3156001

[ref14] Fredrickson B. L. (2001). The role of positive emotions in positive psychology: the broaden-and-build theory of positive emotions. Am. Psychol. 56, 218–226. doi: 10.1037/0003-066X.56.3.218, 11315248 PMC3122271

[ref15] Guida J. L. Alfini A. Lee K. C. Miller C. Riscuta G. Rusch H. L. . (2023). Integrating sleep health into resilience research. Stress. Health 39, 22–27. doi: 10.1002/smi.3244, 36976713

[ref16] Ha Y. Q. Li Q. Ma K. Zhang J. S. Song Y. T. Zhang J. (2024). Effect of Guiyuan Formula on Taiyin Constitution Personality and Cognitive Function in Elderly Patients with Yang-Deficiency Insomnia. Available online at: https://kns.cnki.net/kcms2/article/abstract?v= (Accessed February 1, 2026)

[ref17] Han X. Yang H. F. (2009). A trial application of the Nolen-Hoeksema rumination response scale in China. Chin. J. Clin. Psychol. 17, 550–551. doi: 10.16128/j.cnki.1005-3611.2009.05.028

[ref18] Jiang P. K. Huang H. Zhan H. X. Zhong X. C. Cheng L. H. Xu Z. P. . (2023). The mediation effect of psychological resilience on the relationship between sleep quality and perceived stress among staff in a specific unit. World J. Sleep Med. 10, 2142–2144.

[ref19] Lazarus R. S. Folkman S. (1984). Stress, Appraisal, and Coping. New York: Springer Publishing Company.

[ref20] Li Q. Ding C. (2013). A study on the relationship between five-pattern personality and mental health among undergraduates in a college of traditional Chinese medicine. J. Hubei Univ. National. 30, 68–70.

[ref21] Li Y. Y. Li L. Zhu X. X. Huang S. Zhang L. F. (2021). Analysis of the current status and influencing factors of psychological resilience in patients with primary liver cancer. Nurs. Pract. Res. 18, 993–998.

[ref22] Li Y. R. Liu J. Y. (2023). The influence of family cultural capital on primary school students' mental health. Campus Psychol. 21, 159–164. doi: 10.19521/j.cnki.1673-1662.2023.03.002

[ref23] Li S. Mai Q. Mei X. Jiang Y. Xiong Y. Zeng Y. . (2025). The longitudinal association between resilience and sleep quality in breast cancer. Eur. J. Oncol. Nurs. 74:102734. doi: 10.1016/j.ejon.2024.102734, 39571333

[ref24] Liang T. (2025). A Study on the Relationship between Perceived Stress, Rumination, and sleep Quality in Advanced Pregnant Women during the Third Trimester [Master's thesis]. Nanning: Guangxi University of Chinese Medicine.

[ref25] Liu X. C. Tang M. Q. Hu L. Wang A. Z. Wu H. X. Zhao G. F. . (1996). A study on the reliability and validity of the Pittsburgh sleep quality index. Chin. J. Psychiatry 2, 103–107.

[ref26] Liu Y. A. Yang X. W. Li L. Z. (2022). A study on the correlation between the five-pattern personality and psychological resilience among nursing interns. China Mod. Med. 29, 152–155.

[ref27] Lu M. H. Ya S. X. (2016). Research on the theory of temperament and the five-pattern personality Proceedings of the 15th National Academic Conference of the Psychiatric Professional Committee of Chinese Association of Integrative Medicine 149–154.

[ref28] Masten A. S. (2009). Ordinary magic: lessons from research on resilience in human d development. Educ. Canada 49, 28–32.

[ref29] Nolen-Hoeksema S. Wisco B. E. Lyubomirsky S. (2008). Rethinking rumination. Perspect. Psychol. Sci. 3, 400–424. doi: 10.1111/j.1745-6924.2008.00088.x, 26158958

[ref31] Park C. L. Folkman S. (1997). Meaning in the context of stress and coping. Rev. Gen. Psychol. 1, 115–144. doi: 10.1037/1089-2680.1.2.115

[ref32] Pilcher J. J. Huffcutt A. I. (1996). Effects of sleep deprivation on performance: a meta-analysis. Sleep 19, 318–326. doi: 10.1093/sleep/19.4.318, 8776790

[ref33] Podsakoff P. M. MacKenzie S. B. Lee J.-Y. Podsakoff N. P. (2003). Common method biases in behavioral research: a critical review of the literature and recommended remedies. J. Appl. Psychol. 88, 879–903. doi: 10.1037/0021-9010.88.5.879, 14516251

[ref34] Podsakoff P. M. MacKenzie S. B. Podsakoff N. P. (2012). Sources of method bias in social science research and recommendations on how to control it. Annu. Rev. Psychol. 63, 539–569. doi: 10.1146/annurev-psych-120710-100452 21838546

[ref35] Smith B. W. Dalen J. Wiggins K. Tooley E. Christopher P. Bernard J. (2008). The brief resilience scale: assessing the ability to bounce back. Int. J. Behav. Med. 15, 194–200. doi: 10.1080/10705500802222972, 18696313

[ref36] Southwick S. M. Charney D. S. (2012). The science of resilience: implications for the prevention and treatment of depression. Science 338, 79–82. doi: 10.1126/science.1222942, 23042887

[ref37] Sun Y. Q. Zou Q. F. Zhang B. X. Liao X. Y. Qiu D. R. (2025). The mediation effect of psychological resilience between personality traits and career adaptability in nursing interns. Occup. Health 41, 2695–2701. doi: 10.13329/j.cnki.zyyjk.2025.0465

[ref38] Tang Y. S. (2015). A Study on the Correlation between Insomnia and the Five-Pattern Personality in Traditional Chinese medicine and Stressful life Events [Master's thesis]. Guangzhou: Guangzhou University of Chinese Medicine.

[ref39] Tousignant O. H. Taylor N. D. Suvak M. K. Fireman G. D. (2019). Effects of rumination and worry on sleep. Behav. Ther. 50, 558–570. doi: 10.1016/j.beth.2018.09.005, 31030873

[ref40] Tugade M. M. Fredrickson B. L. (2004). Resilient individuals use positive emotions to bounce back from negative emotional experiences. J. Pers. Soc. Psychol. 86, 320–333. doi: 10.1037/0022-3514.86.2.320, 14769087 PMC3132556

[ref41] Yang Q. L. Xue C. C. (2006). The personality theory of traditional Chinese medical psychology and the five-pattern personality test. Chin. J. Basic Med. Tradit. Chin. Med. 10, 777–779.

[ref42] Yu X. N. Zhang J. X. (2007). A comparative study on the application of the self-resilience scale and the Connor-Davidson resilience scale. Psychol. Sci. 5, 1169–1171. doi: 10.16719/j.cnki.1671-6981.2007.05.035

[ref43] Zhang D. B. (2009). Research on the theory of yin and yang in Huangdi Neijing. J. Shanxi Coll. Tradit. Chin. Med. 10, 2–8.

[ref44] Zhang D. Hu X. Q. Liu Q. X. (2021). Stress and sleep quality among college students: the chain mediation effect of rumination and psychological resilience. Psychol. Sci. 44, 90–96. doi: 10.16719/j.cnki.1671-6981.20210113

[ref45] Zheng Z. H. Qi W. J. Guo X. Q. (2025). The impact of negative life events on sleep in college students: the mediation effect of rumination. Psychology Monthly 20, 119–122. doi: 10.19738/j.cnki.psy.2025.21.033

[ref46] Zhou N. N. (2011). A Study on the Correlation between Psychological Characteristics, Traditional Chinese Medicine Personality and Constitution, and TCM Syndrome Patterns in Patients with Insomnia [Master's thesis]. Beijing: Beijing University of Chinese Medicine.

