# Correction: Association of Taiyin personality traits with sleep quality among college students: parallel indirect effects through resilience and rumination

**DOI:** 10.3389/fpsyg.2026.2029634

**Published:** 2026-10-26

**Authors:** Guanghua Pan, Rui Zhang, Xiaokang Wang, Yuelin Zhai, Anran Wang, Haijuan Zhu

**Affiliations:** 1 College of Health Science, Shandong University of Traditional Chinese Medicine, Jinan, China; 2 Student Affairs Department, Shandong University of Finance and Economics, Jinan, Shandong, China; 3 School of Teacher Education, Qilu Normal University, Jinan, China

**Keywords:** parallel mediation, psychological resilience, ruminative thinking, sleep quality, Taiyin personality

Authors Guanghua Pan and Rui Zhang were erroneously listed as corresponding authors.

The corresponding author is Haijuan Zhu, email: yzyjh2008@sina.com.

The equal contribution statement was missing from authors Guanghua Pan and Rui Zhang.

^†^These authors have contributed equally to this work.

The original version of this article has been updated.

